# ReGeneraTing Agents (rgta^®^) technology combined with antibiotics improves outcomes for infections in the upper limb

**DOI:** 10.1002/ccr3.3645

**Published:** 2021-01-27

**Authors:** Sharifah Ahmad Roohi, Zela Keuylian, Denis Barritault

**Affiliations:** ^1^ Hand & Upper Limb Centre Pantai Hospital Kuala Lumpur Kuala Lumpur Malaysia; ^2^ Prince Court Medical Centre Orthopaedic Clinic Kuala Lumpur Malaysia; ^3^ OTR3 Paris France; ^4^ Laboratory Cell Growth and Tissue Repair (CRRET) UPEC 4397/ERL CNRS 9215 Université‐Paris‐Est‐Créteil Créteil France

**Keywords:** CACIPLIQ20®, heparan sulfate mimetic, infection, matrix therapy, wound healing

## Abstract

A matrix therapy agent marketed as CACIPLIQ20^®^ showed marked improvement in the healing rate of hand infections, including functional recovery. It can be used at both earlier and later stages to promote faster healing and prevent an adverse outcome.

## Graphical abstract

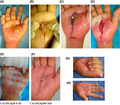

## 1 INTRODUCTION

In this case series, four patients developed or presented with severe infections. In addition to antimicrobial therapy, they were treated with a new matrix therapy technology called CACIPLIQ20^®^. CACIPLIQ20^®^ markedly improved the outcomes of all cases in terms of healing times and range of motion despite healing by secondary intention.

Infection in orthopedic trauma patients is common and associated with substantial financial and psychosocial costs which can decrease quality of life. Despite various strategies used to prevent infection (ie, use of antibiotic‐impregnated implant coatings and cement), managing infections and achieving a satisfactory and predictable outcome remain challenging.^1^ The infection rate in orthopedics, depending on the location and severity of the injury, could range from 5% to 10% for acute trauma cases.^1^ In patients with diabetes, this rate doubles, while in patients with high blood sugar at the time of presentation, infection rates may reach up to 32%.^2^ An open, infected wound compromises therapy—even with stable fixation, for it may lead to the formation of scar tissue in postinfection healing, resulting in contractures and stiffness.

Here, we had the opportunity to use a novel matrix therapy agent in the field of regenerative medicine, called CACIPLIQ20^®^, also known as a RGTA^®^ (ReGeneraTing Agent). RGTAs^®^ are heparan sulfate (HS) mimetics which use a minimally invasive approach to promote tissue regeneration by reconstructing the cellular microenvironment following tissue injuries.^3^ CACIPLIQ20^®^ contains OTR4120, a specifically designed RGTA® formulated for skin and plastic surgery.^4^, ^5^ It has also been recommended to treat chronic wounds in diabetic patients.^6^, ^7^ In orthopedics, hand, and microsurgery, it has been shown to improve clinical outcomes when used to treat wounds resulting from amputations and burns.^8^, ^9^

Here, we present a series of four cases who either already presented with or later developed infections on their hands and/or fingers. CACIPLIQ20^®^ treatment was initially used as a last resort to avoid an undesirable outcome and then progressively used at earlier stages to improve functionality.

## 2 METHODS

### 2.1 Patients

All four cases were chosen after a specific surgical index procedure was performed in the emergency setting where the wounds were either present primarily or developed secondarily due to failure of the conventional treatment (debridement, antibiotics, and dressing) in the primary setting. CACIPLIQ20^®^ was applied (as described below) when the wounds were not healing or were regressing. The clinical data are summarized in Table 1.

**TABLE 1:** Summary of the amputation cases (n = 7)

Case #	Age/gender	Type of lesion	Co‐morbidity	Primary or secondary intention & history	Ischemia (Y/N)	Start of CCPL (days)	Estimated success evaluation
1	57/F	Palmar abscess	DM	2nd, after IV AB treatment, then local gentamycin	N	POD12	Quicker full recovery
2	69/M	Right MF Extensor tenosynovitis	None	2nd, incision and drainage failure of oral AB, IV ciprofloxacin	N	POD6	Speedy healing and full functional recovery of tendon
3	24/M	IF Cellulitis/tenosynovitis	None	1st, incision and drainage starting necrosis. Revascularization and reversion of necrosis	N	POD7 4 days after first sign of flap tip necrosis	revascularization and reversion of necrosis within 4 days and ultimately full healing
4	54/M	R SF flap & R hand incision, infection	DM, renal impairment	2nd, necrosis of flap and secondary infection, SSI of 2nd metacarpal fixation	N	POD14, 1 week after infection	3 months until complete healing

Note

AB: antibiotic; CCPL: CACIPLIQ20®; DM: diabetes mellitus; IF: index finger; IV: intravenous; MF: middle finger; POD: after surgery; SF: small finger; SSI: surgical site infection

### 2.2 Materials

CACIPLIQ20® is the commercial name for a RGTA^®^‐based product used to treat chronic wounds with loss of skin and subcutaneous tissue. RGTA^®^s are heparan sulfate (HS) mimetics, specifically designed to replace degraded HS in damaged tissue, accelerating the speed and enhancing the quality of tissue repair.^3^ Their unique properties have been the subject of intensive preclinical and clinical studies.^5^, ^6^, ^7^, ^10^, ^11^, ^12^, ^13^, ^14^, ^15^ CACIPLIQ20^®^ contains RGTA^®^ OTR4120, a biodegradable α‐1‐6‐carboxymethylsulfated polyglucose polymer.^3^

In normal tissue, the extracellular matrix (ECM) scaffold, made up of structural proteins (ie, collagen, fibronectin, and laminin) and communication peptides are held together by HS molecules. In injured tissue, HS present at the cell surface and within the extracellular matrix (ECM) is degraded by heparinase; this exposes other ECM components, which are required for wound healing and homeostasis, to be destroyed by locally secreted proteases, breaking down this essential scaffold.^16^ One of the most significant properties of RGTAs^®^ is their resistance to glycanase degradation, while being structurally and functionally analogous to naturally derived HS. Therefore, in the microenvironment of chronic wounds which are characterized by unrestrained proteolytic activity, this unique property allows RGTAs^®^ to retain their structure and activity.^3^, ^12^, ^16^ Thus, they are able to replace the destroyed HS, restore the microenvironment, and foster tissue healing. By restoring the natural architecture of the ECM and binding to free “heparan‐binding sites” present in structural proteins such as collagen, fibronectin, and laminin,^12^ RGTA^®^ protects the matrix proteins from proteolytic degradation and facilitates reconstruction of the ECM scaffold, a necessary first step in re‐establishing a microenvironment conducive to tissue repair.^16^, ^17^

A second feature of chronic wounds is a reduction in the levels of growth factors required for matrix formation, remodeling, formulation of granulation tissue, and re‐epithelialization due to the high levels of proteolytic enzymes released by inflammatory cells. A notable feature of RGTAs^®^ is that they are able to protect and potentiate signaling peptides and growth factors, thereby re‐establishing the ECM communication network.^18^ RGTAs^®^ are able to bind numerous heparin‐binding growth factors including FGF,^19^ VEGF^12^ and TGFβ,^19^ or chemokines such as SDF‐1^20^ and, in doing so, protect them from proteolytic degradation and increase their bioavailability.^21^ By re‐establishing the spatiotemporal growth factor distribution, RGTAs^®^ may influence important processes contributing to tissue healing and regeneration such as angiogenesis, cell migration, and differentiation.^12^, ^14^, ^20^, ^22^, ^23^, ^24^

RGTAs^®^ are biodegraded when internalized and catabolized through lysosomal pathways within cells like any other matrix element. The turnover of matrix constituents, which is tissue specific, is also dependent on the extent of the injury. The extent of RGTA^®^‐induced restoration depends on the dose and frequency of its use. Therefore, dosing and timing should be adapted to the tissue and to the state of the injury.

### 2.3 Intervention

Twice‐weekly application of a sterile gauze soaked with CACIPLIQ20^®^ was placed on the wound for 12 minutes and then removed. The wound was then covered with a nonocclusive dressing. Debridement, local antibiotics, and appropriate dressing materials were substituted or used in conjunction before or after the intervention as required.

## 3 RESULTS

### 3.1 Case 1 Diabetic Palmar Abscess

**FIGURE 1:**
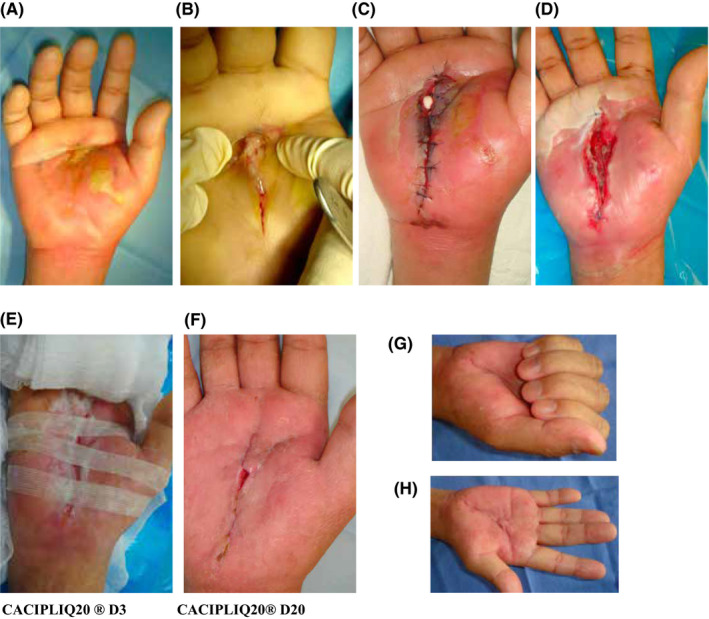
A, Pockets of pus with surrounding areas of ascending inflammation. B, Incision and drainage. C, An inflamed wound requiring local antibiotics. D, The wound has improved but is deep and wide, measuring 40 x 60 mm. E, CACIPLIQ20^®^ was started and wide steri‐strips were used to strap the hand transversely to help approximate the wound edges. F, 20 days later, a small gap remains, and the wound is almost healed. G, Full fist flexion and H, full extension of the digits

Case 1, a 57‐year‐old diabetic female presenting with a palmar abscess of her right hand, was referred after 5 days of hospitalization and failed treatment with intravenous antibiotics. There was swelling of both the mid‐palmar space and the thenar eminence (Figure 1A). She was taken for incision and drainage after magnetic resonance imaging (MRI) confirmed a dorsal swelling as well as a 5 cm abscess in the palm (Figure 1B). Immediate surgical intervention was performed, 2cc of frank pus was aspirated and sent for culture. The wound was partially closed and monitored closely. The following day, the inflammation had not resolved, so alternate stitches were removed and local gentamycin antibiotic beads were embedded in the palmar wound (Figure 1C). Within a week, the erythema had reduced, the wound was granulating and looked very clean; however, the remaining gap was large (40 mm by 60 mm) (Figure 1D). It was decided to use CACIPLIQ20^®^ to assist wound closure while bands of steri‐strip were applied instead of sutures to help approximate the wound edges (Figure 1E). By 20 days, a small gap remained which went on to unite (Figure 1F). Constant therapy by the nurse or hand therapist during dressings helped to maintain a full range of motion and functionality of the hand (grip and pinch), which are essential parts of treatment (Figure 1G, H).

#### 3.1.1 Discussion

Case 1 presented a clean, deep‐pocketed diabetic palmar wound measuring W40 x L60 x D7 mm. CACIPLIQ20^®^ was started 12 days later after the initial debridement and antibiotic treatment (Figure 1E). Within 2 days, the pocket was filled with granulation tissue and had contracted and within 3 weeks the wound was almost closed (Figure 1F). The rapidity of healing seen here, especially in a diabetic, as well as the flexibility and movement maintained in the hand and fingers (which would usually be markedly reduced or lost) and the lack of contracture (Figure 1G, H) are all factors which we attributed to the regenerative agent.

### 3.2 Case 2 Extensor Tenosynovitis

Case 2, a 69‐year‐old right hand‐dominant consultant, noted a small blister over the dorsal tip of his right middle finger (RMF) on his arrival in Malaysia for some business. He took some oral antibiotics from the local practitioner. Two days later, it became more inflamed and he went to the same practitioner who made an incision over the small pustule. Not satisfied with the resulting wound, he came to the hospital emergency room 5 days after his first presentation and was seen by the Medical Officer, who promptly referred him to our practice the following morning. The patient did not have any past history of infection nor family history of diabetes.

**FIGURE 2:**
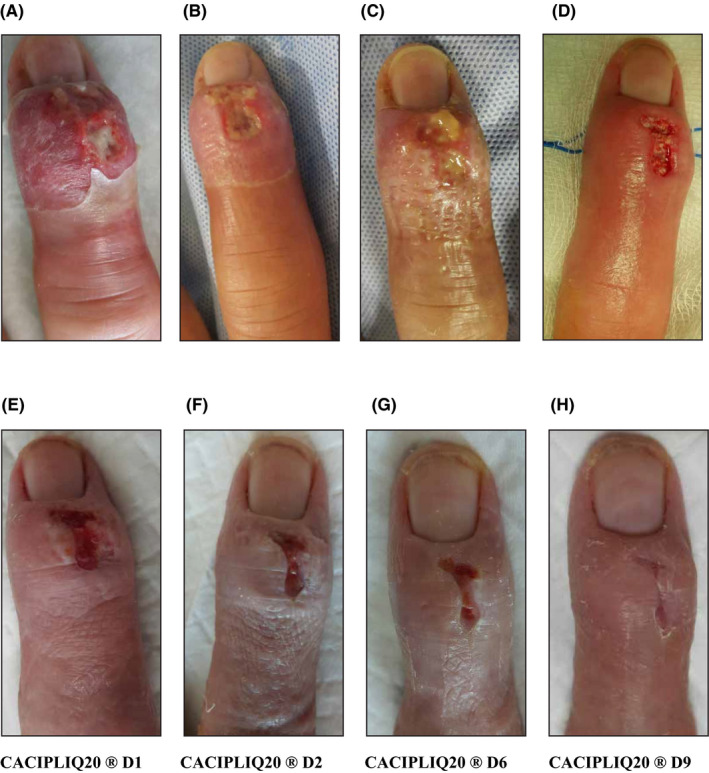
A, Highly erythematous digit with inflammation extending proximally. B, After 2 days of antibiotics, the inflammation and erythema reduced but the wound was still dirty. C, Another 2 days later, the wound was not improving. D, He was taken to theater and debrided thoroughly. E, Two days after debridement, the results were not as promising and CACIPLIQ20® was started. F, An immediate reduction in the width of the wound was noted and followed by the depth—bridging granulation seen (G). H, Final appearance showing the returning shape of the finger

On examination, the right MF was extremely swollen (twice the size of the left MF), tender to the touch, and had a mallet deformity (Figure 2A). There was proximal inflammation up to the mid‐metacarpal level on the dorsal surface of the hand with mild tenderness. The patient's temperature and vital signs were normal. Bloodwork was sent to the laboratory and a Diagnosis of R MF Extensor Tenosynovitis with secondary mallet deformity was made. He was advised admission for IV antibiotics and possible debridement.

The X‐rays showed no signs of osteomyelitis. TW was 6.1 x 10^9^/L with slight eosinophilia (7%), ESR was 23mm/Hr, glucose 5.1mmol/L, uric acid 0.27mmol/L, albumin / proteins were low (30/63 g/L), and CRP was very high at 41.8mg/L.

After 2 days of IV antibiotics (Ciprofloxacin) and daily dressings, the proximal erythema had reduced dramatically but the distal fingertip was still quite swollen. The dorsal wound had an exudative discharge and persistent slough underneath it (Figure 2B). A decision to operate was made, and the patient underwent debridement and curettage of the slough underneath the wound on the 4th day after admission (Figure 2C‐D). Intraoperatively, it was found that the extensor tendon had eroded off its distal insertion causing the mallet. The wound slightly improved 2 days postsurgery but not as much as expected. Since the patient was pressed for time, CACIPLIQ20^®^ treatment was started with the prospect of accelerating wound healing (Figure 2E). Within 2 days, a dramatic improvement was seen with the two edges approximating (Figure 2F) and in another 4 days the wound almost closed (Figure 2G). The last day of follow‐up (3 days later) showed closure of the wound (Figure 2H). The patient was discharged with a splint in extension, and the oral antibiotics were also stopped. A month later, the patient informed us that his extensor tendon had healed.

#### 3.2.1 Discussion

Case 2 was an expatriate with a deadline to return home. The wound was showing marginal improvement; thus, CACIPLIQ20^®^ was started 2 days after debridement (Figure 2E). Although the wound size was small, a dramatic change was seen both in the depth and width of the wound margins, with closure being achieved in 7 days (Figure 2G). The extensor tendon may have also benefited from the regenerating agent because one month is a relatively short period for an avulsion to heal; however, we could not obtain any supporting evidence.

### 3.3 Case 3 Cellulitis / tip of skin‐flap necrosis

**FIGURE 3:**
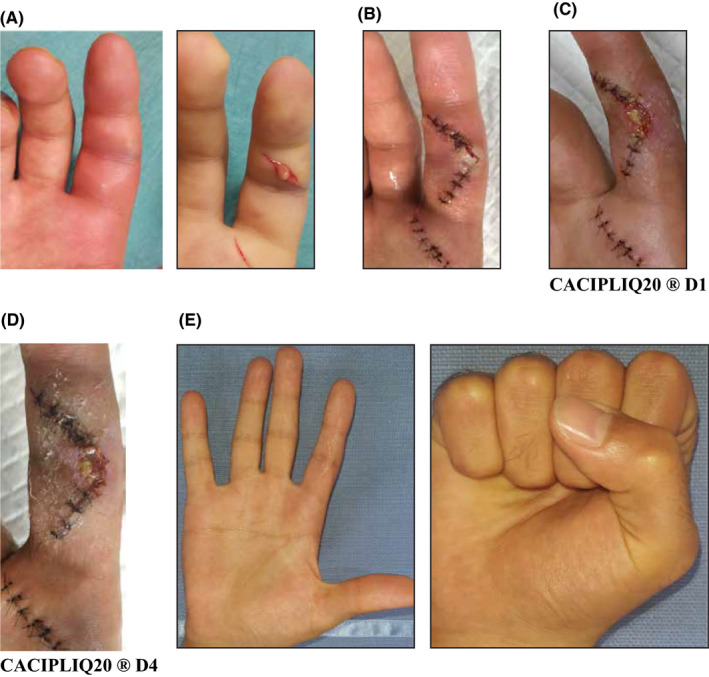
A, left panel There were obvious signs of inflammation; an incision was made showing pus discharge which was limited to the site (A right panel). B, The skin flap showing early ischemia, POD 3. C, CACIPLIQ20® was applied on POD 8. Small area of slough seen. D, Flap edge revascularized and slough decreased within 3 days (POD 11). E, Full range of motion achieved

Case 3, a right hand‐dominant 24‐year‐old creative designer, presented with an inflamed Right Index Finger (IF) of 4 days’ duration which he developed after he poked and peeled off a long‐existing epidermal cyst near the middle crease of his IF (Figure 3A). The finger appeared to be a flexor tenosynovitis, and he was immediately advised drainage. A Bruner's incision with an obtuse‐angled skin flap ultimately led to full‐thickness necrosis at the tip which sloughed off (Figure 3B). CACIPLIQ20® was applied on POD8 (Figure 3C). Within 3 days of application of the regenerative agent, revascularization could be seen and the slough reduced (Figure 3D). Within a week, the wound had healed. On the final follow‐up 3 months later, full range of motion (ROM) was achieved with good scar healing (Figure 3E).

#### 3.3.1 Discussion

Early application of the regenerative agent was found to be extremely effective at stimulating blood supply. Although a very small area of ischemia, the obtuse angle of the skin flap should not have resulted in ischemia; thus, it was red flagged and prompt action was warranted. Again, suppleness of skin in areas of skin creases was seen, which is not the case in healing by secondary intention where scarring results in contracture.

### 3.4 Case 4 Infection of traumatic wound site and surgical site infection

**FIGURE 4:**
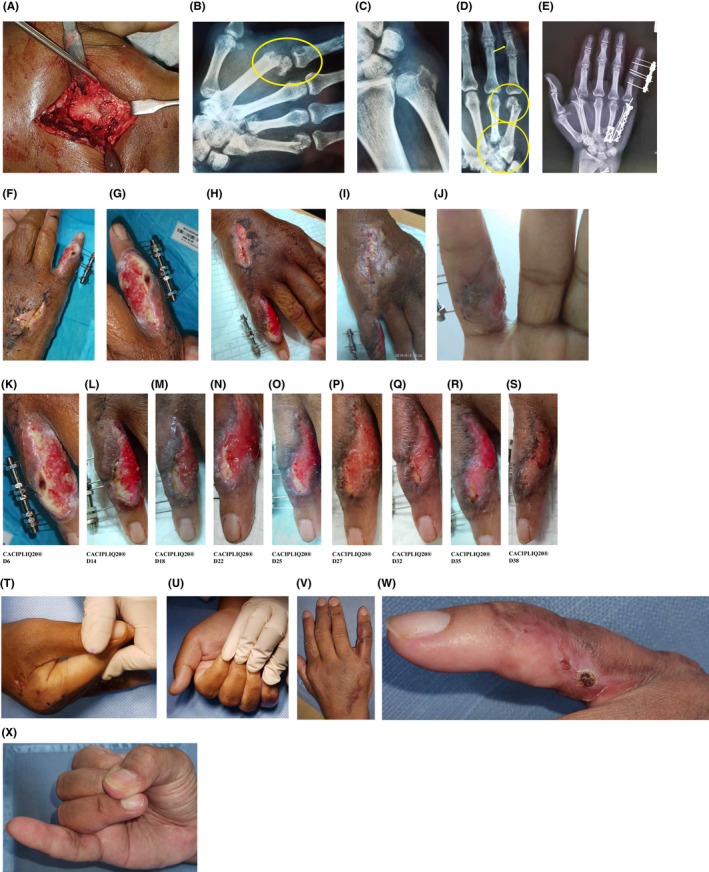
A‐D, Fixation of an open middle phalanx (P2) fracture with a concomitant degloving injury and avulsion flap of the small finger, closed 2nd and 5th metacarpal (MC) neck fractures, 4th and 5th metacarpal base fractures with dislocation of both 4th and 5th carpo‐metacarpal (CMC) joints and an Ulna styloid base fracture (yellow arrow and circles in the figures). Intraoperative fixation was prolonged and made difficult by the brittleness of the patient's bones and the lytic lesions created by the tophi. E The postoperative radiograph showed good fixation. F‐G, The wounds started to break down when blood sugar was no longer controlled. Multiple infections were found, so the dressing was changed and the wound was sprayed with Cacipliq at *POD14*. H‐I, Desloughing during dressings was performed every other day, and local application of Fucidin cream was commenced, improving the wound's status. J, Within 6 days, the wound was drier and seemed a little contracted, with healing of the volar wound as well. K‐S, The SF wound took a total of 38 days to dry up. T‐U, The patient was then taken to the theater for removal of all implants and manipulation under anesthesia. V‐X, The patient lost small finger motion but retained index finger ROM as well as its use

Case 4, a 54‐year‐old right hand‐dominant company director, had a motorcycle accident and sustained multiple fractures (six) and dislocations (two) of his (dominant) right hand and wrist. At presentation, he had uncontrolled diabetes (RBS 24.6 mmol/L) and long‐standing gout with a skyrocketing uric acid level of 699 µmol/L (normal range is 204‐420 µmol/L) and mild renal impairment. He was taken into the operation theater after stabilization of his blood sugars that same evening for fixation of: an open middle phalanx (P2) fracture with a concomitant degloving injury and avulsion flap of the small finger, closed 2nd and 5th metacarpal (MC) neck fractures, 4th and 5th metacarpal base fractures with dislocation of both 4th and 5th carpo‐metacarpal (CMC) joints, and an Ulna styloid base fracture. Perioperative blood sugars were reasonably controlled, but intraoperative fixation was prolonged and made difficult by the brittleness of his bones and the lytic lesions created by the tophi (Figure 4A‐D), especially the 2nd metacarpal head which could only be fixed with wires. The postoperative radiograph (Figure 4E) showed good fixation.

He had a first dressing change on the third postoperative day by which time his blood sugar wase halved, but the uric acid was still high. Uric acid only decreased to a reasonable level (444 µmol/L) on POD5, and the patient was subsequently discharged. On the 2nd postoperative dressing a week later (POD12), the wounds on the small finger were infected and the degloved area partially necrosed. Debridement was completed, the area was washed with normal saline, and a Mepitel® dressing was applied. Two days later, the surgical incision for the 4th and 5th MC approach was also found to be infected, so the dressing was changed and the wound was sprayed with CACIPLIQ20^®^ (Figure 4F‐G CCP D1 = POD14). On follow‐up 5 days later, the wound had to be further desloughed. The patient's blood sugar was still elevated at 10 mmol/L. Desloughing during dressings were performed every other day, and local application of Fucidin cream was commenced, improving the wound's status (Figure 4H‐I). A swab was taken and cultured *Pseudomonas aeruginosa,* so the antibiotic was later changed to Ciprofloxacin. Within 6 days, the wound was dried and seemed a little contracted, with healing of the volar wound as well (Figure 4J). The principal challenge was the SF wound: It took a total of 38 days to dry up (Figure 4K‐S) during which time the fractures simultaneously healed. The patient was then taken to the theater for removal of all implants and manipulation under anesthesia (Figure 4T‐4U). Although this technique is extremely useful, one must accept that there will be some loss of the intraoperative results achieved (Figure 4V‐X).

#### 3.4.1 Discussion

Healing is often negatively affected in diabetic patients with renal impairment, especially if blood sugar is not well controlled. Once infection sets in, it becomes an uphill battle to eradicate it and maintain skeletal stability. A good way to achieve this is through external fixation of open fractures, frequent wound inspections, and keeping tight control of blood sugar levels with appropriate antibiotic (local and systemic) administration. The infection in this patient was controlled once his blood sugar levels dropped and the antibiotic choice was appropriate. CACIPLIQ20^®^ was effective only after blood sugar was controlled (Figure 4M‐S). This brings an important point for discussion—the basic factors to control infection must be implemented in order for CACIPLIQ20^®^ to be effective. One must also learn to predict which factors may prompt or lead to infection. Wise et al devised a scoring system which predicts postoperative surgical site infection.^25^ The use of this predictive scoring system would greatly enhance the surgeon's ability to preempt potential factors and to also try and overcome the ones that are already present.

## 4 SUMMARY OF RESULTS

All the wounds in the four presented cases healed successfully. Interestingly, pain was reduced dramatically with each application. Two‐point discrimination was restored to normal levels in two patients with flaps. No adverse events were recorded. All patients returned to their previous job or activity level with minimal functional deficit.

## 5 GENERAL DISCUSSION

Regulation of cellular proliferation plays a significant role in many pathologies and has been thoroughly studied. Two key elements that aid wound healing are vascular supply and elimination of infection. Castellot et al described how heparin acts as a bifunctional (up and down) regulator of smooth muscle cell growth in the vascular endothelium.^26^ Olczyk in 2015 described the diverse roles of HS in skin wound healing and said the implementation of new therapeutic treatment strategies was promising.^27^ While the restorative capacity of glycosaminoglycans is impressive, other sulfated glycosaminoglycans such as chondroitin sulfates (A, B, or C) do not share most of the binding and protection capabilities of matrix proteins or communication peptides like heparan sulfates do.^16^, ^27^ In contrast, some other products substitute the entire scaffold (extracellular matrix components, cross linked proteins and growth factors, etc) and then induce seeding or colonization of cells, which is a two‐step process.

In these case studies, we show that RGTA^®^ therapy resulted in successful wound healing by secondary intention in all four infection cases and was able to restore a full recovery of the injured tissue despite the presence of infection. Added benefits included little or no scarring with suppleness allowing greater mobility over joints, even with healing by secondary intention. Moreover, the pain score was also reduced with this intervention, although not recorded in all patients. Further studies to assess the effect on nerve and tendon healing have been described in preclinical motor nerve section or crushed tendon models^28^ and also for tendon lesions in racing horses in a randomized control trial.^29^ This report provides new evidence supporting the clinical potential of this RGTA‐based matrix therapy.

The second interesting property of OTR4120 was its efficacy despite the presence of infection and its effectiveness in combination with antibiotics. This indicates that the presence of bacteria does not necessarily inhibit healing from taking place and that once in motion, the healing process can overcome the infection. This has been shown in two models of periodontitis where partially destroyed maxillary bone and gingival tissues (including cementum) regenerated under treatment with OTR4120 in the presence of an ongoing infection.^30^, ^31^

It is important for all healthcare practitioners who wish to use this product to be aware that thorough debridement of the wound is a prerequisite. Additionally, approximation of tissue aided by supportive tape enhances the process. Furthermore, the use of local and systemic antibiotics is encouraged when deemed necessary. Various dressings of differing frequency may need to be used as an adjunct to deal with the different types of wounds.

As a case report, this manuscript has no other ambition than to inform practitioners of this technology. We would like to highlight the uniqueness of CACIPLIQ20^®^ as a regenerative agent: its ease of use, acceleration of the healing process, replacing like for like tissue and reducing pain, scarring, and contracture. We are not sure of its ability to restore sensation (2‐point discrimination), as it is difficult to prove. (Table 2)

**TABLE 2:** Plus points in favor of usage of CACIPLIQ20^®^

1. Neovascularization develops in ischaemic tissue
2. Regeneration of like for like skin reduces stiffness / contractures
3. Accelerated healing saves cost in long term
4. Helps preserve length of digit—salvage of dying skin graft, flap
5. Reduces hypersensitivity in fingertip amputations
6. Returns 2 P.D to normal in certain injuries
7. Enhances bone and tendon healing
8. Help to maintain / regain shape?

Proper randomized controlled trials would be necessary to assess specific benefits and outcomes, but trials of this nature typically take years to complete and would be costly. Also, it may be challenging to obtain two similar groups of patients with wounds to compare. In conclusion, we feel this case series depicts adequate evidence in support of the use of this RGTA^®^.

## 6 ETHICS APPROVAL

The patients provided informed consent, and the study was approved by the Hospital's institutional review board following the World Medical Association Declaration of Helsinki (June 1964) and subsequent amendments.

## CONFLICT OF INTEREST

DB has financial interest as inventor of patented RGTA technology.

## AUTHOR CONTRIBUTIONS

Authors’ contributions are as follows: RS A: Conception and design of the study; acquisition of data; analysis and interpretation of data. R.SA., DB, and ZK: Writing and revising the article.
